# Roles of GSK-3 and microRNAs on epithelial mesenchymal transition and cancer stem cells

**DOI:** 10.18632/oncotarget.13991

**Published:** 2016-12-16

**Authors:** James A. McCubrey, Timothy L. Fitzgerald, Li V. Yang, Kvin Lertpiriyapong, Linda S. Steelman, Stephen L. Abrams, Giuseppe Montalto, Melchiorre Cervello, Luca M. Neri, Lucio Cocco, Alberto M. Martelli, Piotr Laidler, Joanna Dulińska-Litewka, Dariusz Rakus, Agnieszka Gizak, Ferdinando Nicoletti, Luca Falzone, Saverio Candido, Massimo Libra

**Affiliations:** ^1^ Department of Microbiology and Immunology, Brody School of Medicine at East Carolina University, Greenville, NC, USA; ^2^ Department of Surgery, Brody School of Medicine at East Carolina University, Greenville, NC, USA; ^3^ Department of Internal Medicine, Hematology/Oncology Section, Brody School of Medicine at East Carolina University, Greenville, NC, USA; ^4^ Department of Comparative Medicine, Brody School of Medicine at East Carolina University, Greenville, NC, USA; ^5^ Biomedical Department of Internal Medicine and Specialties, University of Palermo, Palermo, Italy; ^6^ Consiglio Nazionale delle Ricerche, Istituto di Biomedicina e Immunologia Molecolare Alberto Monroy, Palermo, Italy; ^7^ Department of Morphology, Surgery and Experimental Medicine, University of Ferrara, Ferrara, Italy; ^8^ Dipartimento di Scienze Biomediche e Neuromotorie, Universitࠤi Bologna, Bologna, Italy; ^9^ Chair of Medical Biochemistry, Jagiellonian University Medical College, Kraków, Poland; ^10^ Department of Animal Molecular Physiology, Institute of Experimental Biology, Wroclaw University, Wroclaw, Poland; ^11^ Department of Biomedical and Biotechnological Sciences Oncological, Clinical and General Pathology Section, University of Catania, Catania, Italy

**Keywords:** GSK-3, cancer stem cells, Wnt/beta-catenin, PI3K, Akt

## Abstract

Various signaling pathways exert critical roles in the epithelial to mesenchymal transition (EMT) and cancer stem cells (CSCs). The Wnt/beta-catenin, PI3K/PTEN/Akt/mTORC, Ras/Raf/MEK/ERK, hedgehog (Hh), Notch and TP53 pathways elicit essential regulatory influences on cancer initiation, EMT and progression. A common kinase involved in all these pathways is moon-lighting kinase glycogen synthase kinase-3 (GSK-3). These pathways are also regulated by micro-RNAs (miRs). TP53 and components of these pathways can regulate the expression of miRs. Targeting members of these pathways may improve cancer therapy in those malignancies that display their abnormal regulation. This review will discuss the interactions of the multi-functional GSK-3 enzyme in the Wnt/beta-catenin, PI3K/PTEN/Akt/mTORC, Ras/Raf/MEK/ERK, Hh, Notch and TP53 pathways. The regulation of these pathways by miRs and their effects on CSC generation, EMT, invasion and metastasis will be discussed.

## INTRODUCTION

**Figure 1:**
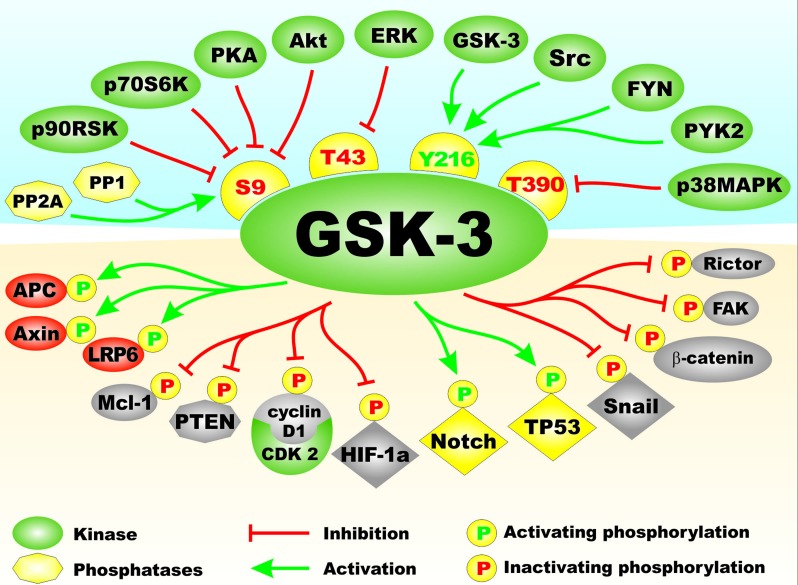
Regulation of GSK-3 Activity by Kinases and Phosphatases and Types of Substrates of GSK-3 On top side of figure above GSK-3 are various kinases which regulate GSK-3. They are depicted in green ovals. Phosphatases which activate GSK-3 are shown in yellow octagons. Amino acid phosphorylation sites which when phosphorylated result in inactivation of GSK are indicated in yellow hemispheres with red letters. The Y216 phosphorylation site which results in activation of GSK-3 is presented in a yellow hemisphere with green letters. Phosphorylation/dephosphorylation events which result in activation of GSK-3 activity are indicated as green arrows. Phosphorylation events which result in inactivation of GSK-3 activity are indicated with red arrows with closed end. On bottom side of the figure below GSK-3 are examples of some of the proteins phosphorylated by GSK-3. Phosphorylation events that result in inactivation are indicated by yellow circles with a red Ps inside. Phosphorylation events that result in activation are indicated by yellow circles with green Ps inside. Types of proteins phosphorylated by GSK-3 include: proteins involved in Wnt/beta-catenin signaling, (*e.g*., APC, Axin, and LRP6) in red ovals as they are activated by GSK-3 phosphorylation or beta-catenin indicated in a grey oval as it is inactivation by GSK-3 phosphorylation. Anti-apoptotic models such as Mcl-1, kinases such as FAK and mTORC components such as Rictor which are inactivated by GSK-3 phosphorylation are indicated in grey ovals. Transcription factors which are activated by GSK-3 phosphorylation are indicated by yellow diamonds. Transcription factors which are inactivated by GSK-3 phosphorylation are indicated by grey diamonds. This figure is presented to provide the reader an idea of the multiple kinases and phosphatases which can regulate GSK-3 on multiple phosphorylation residues and substrates which can be phosphorylated and either inactivated or activated by GSK-3 phosphorylation.

**Figure 2:**
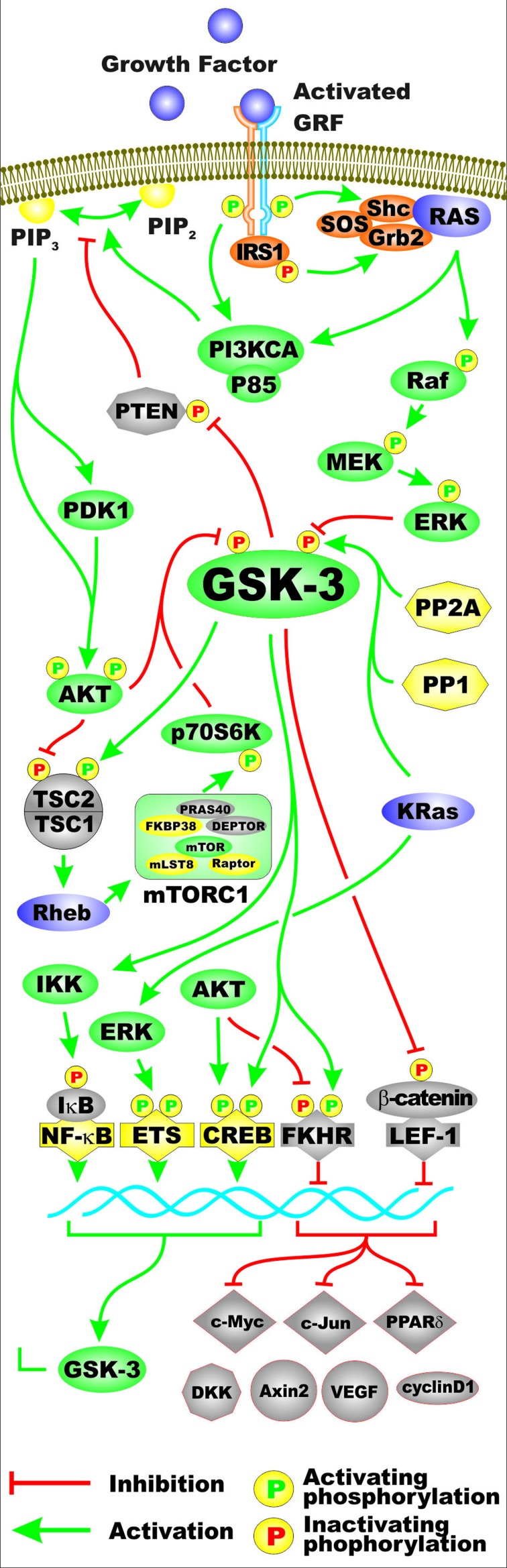
Regulation of GSK-3 Activity by Growth Factor Signaling Two pathways activated by growth factors include the PI3K/PTEN/Akt/mTORC1 and Ras/Raf/MEK/ERK signaling pathways. Some of the regulatory interactions between GSK-3 and PI3K/PTEN/Akt/mTOR and Ras/Raf/MEK/ERK pathways are indicated. A generic growth factor (GF) is indicated in purple and the corresponding growth factor receptor (GFR) are indicated in green and yellow. Ras is indicated by a purple oval. Shc, Grb2 and SOS are indicated in orange ovals. Kinases are indicated in green ovals. The p85 regulatory and the p110 catalytic subunits of PI3K are indicated in green ovals. The PTEN phosphatase which dephosphorylates phosphatidylinositol (3,4,5)-trisphosphate (PIP3) into phosphatidylinositol (4,5)-bisphosphate (PIP2) (yellow ovals) is indicated in a light brown octagon. The PP2A and PP1 phosphatases which may activate GSK-3 by dephosphorylation are indicated in yellow octagons. TSC1 and TSC2 are indicated in light brown semi-circles. mTOR interacting proteins which positively regulate mTOR activity are indicated in yellow ovals. mTOR interacting proteins which negatively regulate mTOR activity are indicated in light brown ovals. Transcription factors activated by either ERK or Akt phosphorylation are indicated in yellow diamonds. The Foxo transcription factor FKHR that is inactivated by Akt phosphorylation is indicated by a light brown diamond. beta-catenin is indicated in light brown oval as it is inactivated after GSK-3 phosphorylation. Some of the types of proteins which are regulated by the beta-catenin pathway are indicated (c-Myc, c-Jun, PPPARδ, DKK, Axin2, VEGF, cyclin D). They are colored in light brown as their expression is inhibited by GSK-3 phosphorylation of beta-catenin. In addition, KRas can induce ERK which can result in activation of Ets and GSK-3 expression which can in turn have effects on IkappaK and NF-kappaB activity. Green arrows indicate activating events in pathways. Red arrows indicate inactivating events in pathway. Activating phosphorylation events are depicted in yellow circles with green Ps inside them. Inactivating phosphorylation events are depicted in yellow circles with red Ps inside them. This figure is provided to give the reader an idea of the multiple interactions of GSK-3 with various signaling molecules in the PI3K/PTEN/Akt/mTOR and Ras/Raf/MEK/ERK pathways.

Glycogen synthase kinase-3 (GSK-3) is moon-lighting kinase which phosphorylates multiple proteins on serine (S) and threonine (T) residues. GSK-3 activity is regulated by many kinases and phosphatases including: the Akt, ERK, FYN, p38^MAPK^, PKA, PYK2 and Src, the protein phosphatases PP1 and PP2A. Phosphorylation of GSK-3 by many of these kinases results in inactivation of GSK-3 activity, while dephosphorylation of GSK-3 by the protein phosphatases often result in activation of GSK-3 activity [1–3]. Figure 1 presents a diagram of the kinases and phosphatases that regulate GSK-3 activity. Also presented in this figure in the bottom portion are some of the different types of substrates phosphorylated by GSK-3. Often when GSK-3 is phosphorylated by some of the above mentioned kinases, it is targeted for protoesomal degradation. Akt is one of the best described regulators of GSK-3. Akt is a component of the PI3K/PTEN/Akt/mTORC1 pathway. Akt is frequently abnormally activated in cancers due to aberrant activity of upstream receptors, activating mutations in the catalytic subunit of PI3K (p110 alpha, *PIK3CA*) or inactivating mutations in the phosphatase and tensin homolog (*PTEN)* lipid and protein phosphatase. Figure 2 presents an overview of the PI3K/PTEN/Akt/mTORC1 and Ras/Raf/MEK/ERK pathways and how they can interact with GSK-3 and regulate its activity. Mutations can occur that result in activation of these pathways and others that will influence GSK-3 activity. The effects of mutations at diverse components of these signaling pathways and sensitivity/resistance to various therapeutics have been recently summarized [4–9].

**Figure 3:**
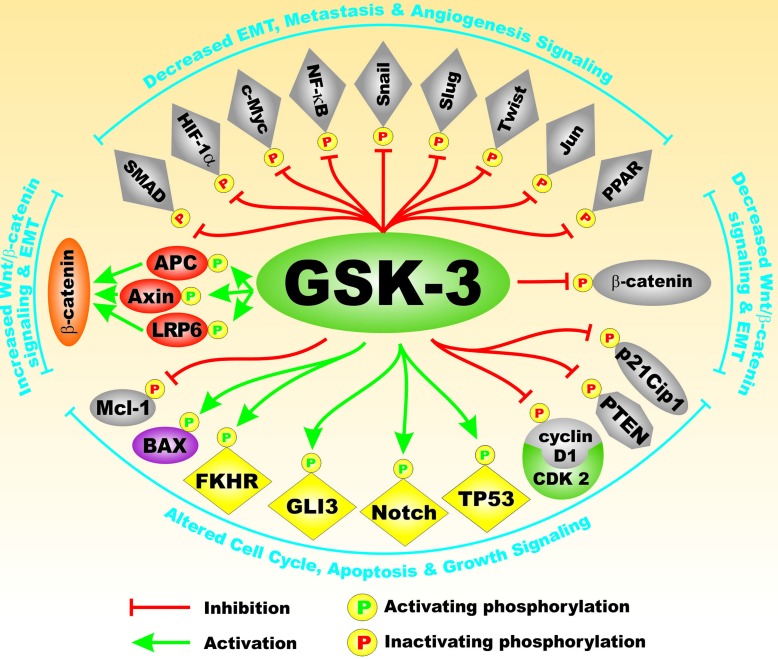
Substrates of GSK-3 which are Involved in EMT On top of GSK-3 are transcription factors which are inactivated by GSK-3 phosphorylation that are indicated in grey diamonds. On bottom of GSK-3 are transcription factors which are activated by GSK-3 phosphorylation that are indicated in yellow diamonds. Indicated in red ovals are molecules in the Wnt/beta-catenin pathway which are activated by GSK-3 phosphorylation which are indicated in red ovals and beta-catenin which is indicated in an orange oval. Also indicated in a maroon oval is the Bax pro-apoptotic molecule. The mitochondrial localization of Bax is promoted when it is phosphorylated by GSK-3 and then it promotes apoptosis. Also indicated are some cell cycle and signaling proteins which are inactivated by GSK-3 phosphorylation (PTEN, Cyclin D1 and p21^Cip-1^) and beta-catenin which are indicated in grey symbols. This figure is presented to provide the reader an idea of the various transcription factors, kinases, phosphatases, apoptotic molecules, cell cycle regulator molecules which are regulated by GSK-3 phosphorylation and may influence EMT.

GSK-3 has many downstream substrates besides glycogen synthase kinase. That is why it is sometimes referred to as a moon-lighting kinase. Some of these proteins play important roles in CSC generation/propagation, invasion and metastasis. Mutations which result in abnormal GSK-3 activity can have effects on the Wnt/beta-catenin pathway and alter the regulation of EMT, and cancer development and metastasis. Figure 3 presents a diagram of some of the types of proteins that GSK-3 interacts with and regulates that are important in EMT. GSK-3 can also interact with and regulate other important developmental pathways implicated in cancer development such as Hh, Gli and Notch. Clearly the moon-lightening kinase GSK-3 has many substrates that perform diverse functions. Even though we often think of signaling pathways as being linear in nature, they are often more spider web in design and altering one component will tweak many additional pathways.

## GSK-3 AND CSCS

**Figure 4:**
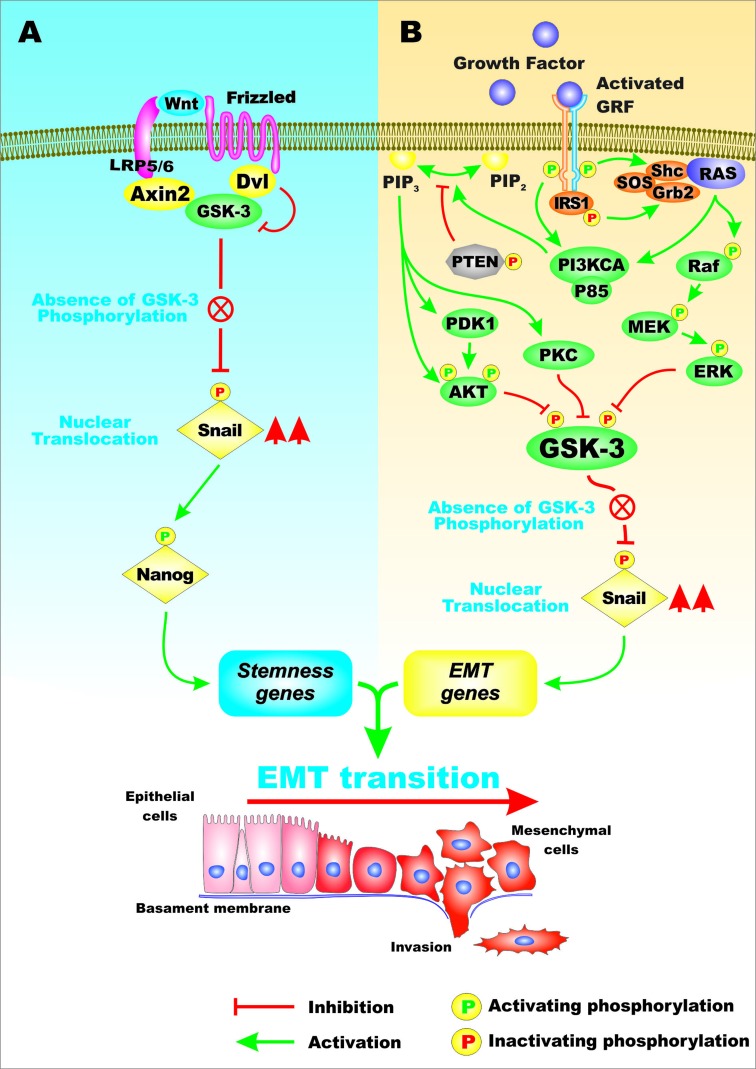
Overview of Regulation of Snail Activity by GSK-3 and Wnt/beta-catenin and Cytokine Medicated Signaling Pathways In Panel **A**., Wnt signaling can prevent GSK-3 phosphorylation which results in the absence of Snail phosphorylation which can have effects on other transcription factors such as Nanog which can regulate the expression of genes involved in EMT. In Panel **B**., cytokine mediated signaling can result in the activation of multiple signaling pathways including PKC, PI3K/PTEN/Akt and Ras/Raf/MEK/REK which can result in GSK-3 phosphorylation that inhibits its activity. In the absence of active GSK-3, Snail is not phosphorylated can have effects on gene involved in EMT. This figure is presented to provide the reader an idea of some of the mechanisms which can alter GSK-3 activity which influence Snail activity and EMT.

The transcription factor Snail is involved in the regulation of gene transcription and is often activated in EMT and metastasis. Snail is important in regulating the expression of the Nanog transcription factor that is important in stemness. The expression and stability of the Snail protein is regulated by GSK-3 at multiple levels. The Wnt/beta-catenin and PI3K/PTEN/Akt/mTORC1 signaling pathways will suppress GSK-3 activity. Figure 4 presents an overview of the effects Wnt/beta-catenin and EGFR mediated signaling on GSK-3, on Snail and EMT.

High levels of Snail have been shown to correlate with metastasis in non-small-cell-lung cancer (NSCLC). Snail-expressing NSCLC cells have some properties present in CSCs. A correlation between Snail expression and phosphorylation of Smad1, Akt, and GSK-3beta was observed in Snail-overexpressing NSCLC cells. The NSCLC CSCs have characteristics of cells that have undergone EMT and were shown to have increased migration, chemoresistance and sphere forming properties. These studies suggest that inactivation of GSK-3beta may be involved in NSCLC CSCs [10].

## ROLES OF WNT/BETA-CATENIN PATHWAY AND GSK-3 IN THE GENERATION AND MALIGNANT PROPERTIES OF CSCS

The earliest description of the involvement of the Wnt/beta-catenin pathway and GSK-3 in CSCs was probably in leukemia stem cells (LSCs) as they were some of the first isolated and characterized CSCs. Certain LSCs have been demonstrated to have higher levels of beta-catenin which also was driving their establishment and drug resistance [2, 11–13].

Other components of the Wnt/beta-catenin signaling pathway may be differentially expressed in certain LSC and other types of CSCs, these include loss of proteins frequently dysregulated in the Wnt/beta-catenin pathway including: functional adenomatous polyposis coli (APC) protein, decreased expression of secreted frizzled-related protein1 (sFRP1), dickkopf-1 (DKK1), increased expression of transcription factor 7 like 2 (TCF7L2), B-cell CLL/lymphoma 9 (BCL9), frizzled class receptor 6 (FZD6), wingless-type MMTV integration site family, member 5A (Wnt5A), Wnt7. Wnt/beta-catenin signaling was determined to be required for self-renewal of not only normal hematopoietic stem cells (HSC) but also LSC.

The Wnt/beta-catenin pathway plays essential roles in colorectal cancers (CRC). This pathway also plays critical roles in CRC CSC [14–17]. Interactions between Wnt and HoxA5 signaling pathways have been observed in CRC. Wnt normally suppresses HoxA5 which maintains stemness. HoxA5 is a repressor of intestinal stem cell fate. A feedback loop between Wnt and HoxA5 signaling has been observed [18]. When HoxA5 signaling becomes active outside the intestinal crypt, Wnt signaling is suppressed and differentiation occurs. HoxA5 signaling is repressed in CRC. However when HoxA5 signaling is induced, loss of the CRC CSC phenotype occurs. Importantly, retenoids can induce HoxA5 that can result in tumor regression [18].

CRC CSCs can be regulated by miRs and large intergenic non-coding RNAs (lncRNAs). The lncRNA-p21 has been determined to be a suppressor of CRC CSCs. lncRNA-p21 inhibited beta-catenin expression [19].

Hypoxia increases the size, CSC-phenotype, and self-renewal properties of CRC CSC cells. This was demonstrated to occur by hypoxia-induced Wnt/beta-catenin signaling and Id2 expression. A Wnt/beta-catenin inhibitor could suppress the levels of CSCs observed after hypoxia treatment and also suppressed the inhibitor of DNA binding 2 (Id2) expression. The Id2 protein inhibits the function of basic helix-loop-helix transcription factors in a dominant-negative (DN) fashion. Suppression of Id2 expression also decreased CRC CSC sphere formation. These studies document a role between Id2 and Wnt/beta catenin expression in CRC CSC formation [20].

## MIRS EFFECTS ON FRIZZLED-RELATED PROTEINS

**Figure 5:**
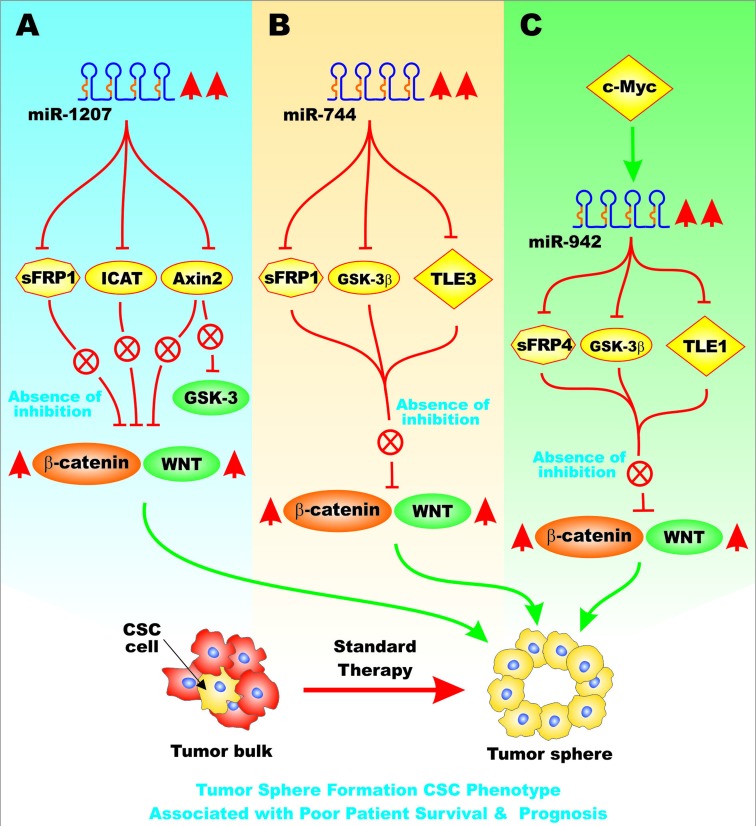
Overview of the Effects of miRs on sFRP Genes and Pathways Involved in EMT Various miRs can influence the expression of key genes involved in EMT. These miRs may display altered regulation in various cancers. In Panel **A**., are presented the effects of miR-1207, that is upregulated in ovarian cancer, on the expression of sFRP1, Axin2 and ICAT mRNAs. These events can result in increased Wnt/beta-catenin signaling that leads to cells with the CSC phenotype. These traits are associated with a poor patient survival in ovarian cancer patients. In Panel **B**., the effects of miR-744 on sFRP1, GSK-3beta, and TLE3 mRNAs are presented that in turn leads to cells with the CSC phenotype, chemotherapeutic drug resistance and is associated with a poor patient prognosis in pancreatic cancer patients. In Panel **C**., the effects of miR-942 on sFRP4, GSK-3beta, and TLE1 mRNAs are presented that can lead to cells with the CSC tumor sphere phenotype and are associated with a poor patient prognosis in esophageal cancer patients. This figure is presented to provide the reader an idea of some of the mechanisms in which miRs can regulate the expression of sFRPs that when inhibited can have effects on EMT and cancer development.

Enforced expression of miR-1207 has been shown to activate the Wnt/beta catenin in ovarian cancer cells with CSC-like properties. This occurred by miR-1207 targeting and suppressing Frizzled-related protein-1 (sFRP1), axin-like protein (Axil) or axis inhibition protein 2 (AXIN2) or conductin, and the inhibitor of beta-catenin and TCF-4 (ICAT). All of these proteins are normally suppressors of the Wnt/beta-catenin pathway. Increased expression of miR-1207 might also alter GSK-3 activity as AXIN2 is suppressed. miR-1207 was determined to be upregulated in ovarian cancer cells and specimens. miR-1207 expression correlated with poor patient overall survival [21]. Figure 5, Panel A, presents a diagram of the effects of miR-1207 on the Wnt/beta-catenin pathway.

miR-744 was determined to affect the expression of the Wnt/beta-catenin pathway in pancreatic cancer and pancreatic spheroid cells. This occurred by miR-744 targeting SFRP1, GSK-3beta and the transducin-like enhancer of Split-3 (TLE3) [22]. miR-744 overexpression also increased the tumorigenicity of the pancreatic cells. miR-744 is believed to be a novel diagnostic and prognostic marker for pancreatic cancer. Overexpression of miR-744 resulted in resistance to gemcitabine *in vitro* [23]. Figure 5, Panel B presents a diagram of the effects of miR-744 on genes involved in CSC phenotype.

miR-942 has been shown to be upregulated in esophageal squamous cell carcinoma (ESCC) and is associated with a poor prognosis in ESCC patients. Increased expression of miR-942 promoted tumor sphere formation. miR-942 was shown to upregulate Wnt/beta-catenin signaling by targeting sFRP4, GSK-3beta and TLE1. These proteins in some cases negatively regulate Wnt/beta-catenin signaling. These studies also demonstrated that c-Myc binds to the miR-942 promoter and stimulates its expression [24]. Figure 5, Panel C presents a diagram of the effects of miR-942 on genes involved in CSC phenotype.

The BCL-2 inhibitor ABT-263 has been shown to synergize with 5-fluorouracil in esophageal cancer. Part of the effects was due to the suppression of many genes involved with stemness as well as inhibition of the Wnt/beta-catenin and YAP/SOX9 axes [25].

**Figure 6:**
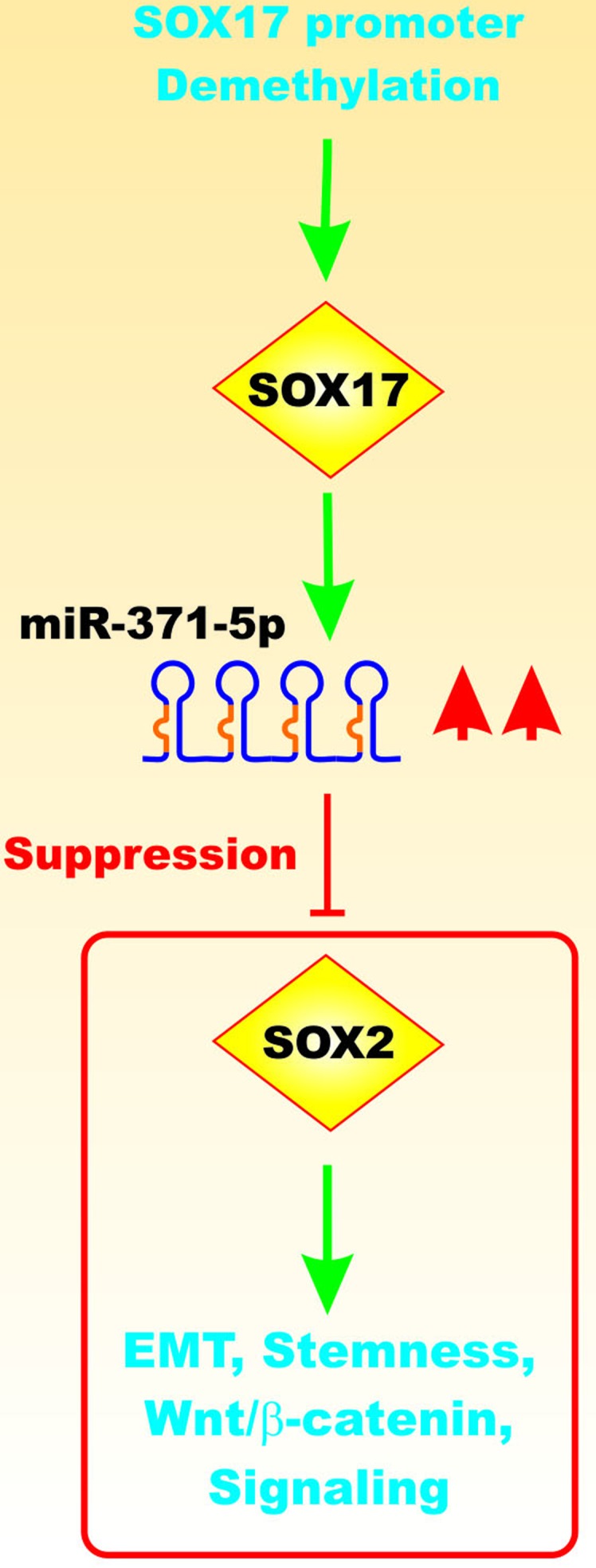
Effects of Sox17 on miR-371-5q Expression and EMT Upon demethylation of the Sox17 gene promoter region, the Sox17 transcription factor is expressed that can induce the transcription of the miR-371-5q miR that can in turn suppress Sox2 and other genes involved in EMT, Wnt/beta-catenin signaling and stemness. This figure is presented to provide the reader an idea of some of the mechanisms by which the Sox17 transcription factor can regulate miRs expression which can regulate in turn the expression of other Sox transcription factors which when inhibited can effects on EMT and cancer development.

miR-371-5p is downregulated in primary CRC tissues compared with matched normal control tissues. miR-371-5p suppressed EMT *via* Wnt-beta catenin signaling. miR-371-5p decreased the CRC stemness phenotype. Demethylation of the Sox17 gene was shown to induce miR-371-5 expression that in turn targeted and suppressed Sox2 expression [26]. Figure 6 presents a diagram of the effects of miR-371-5p on Sox17 expression.

Morphine has been shown to induce Wnt/beta-catenin expression, EMT and metastasis in breast cancer. Nalmefene is an antagonist of morphine and was shown to reverse the effects of morphine. Thus treatment of cancer patients with the pain killer morphine should be critically evaluated [27].

The inflammatory process is important in cancer. Enteric pathogens have been associated with EMT as they may exploit the plasticity of epithelial cells to undergo trans-differentiation. This has been associated with multiple signaling pathways including Wnt, Notch and TGF-beta. In addition, multiple transcription factors including: Slug, Snail, Twist, Zeb1 and Zeb2 may suppress E-cadherin, and effect EMT. Enteric pathogens may alter the EMT pathway and contribute to CSC generation and malignant transformation [28].

**Figure 7:**
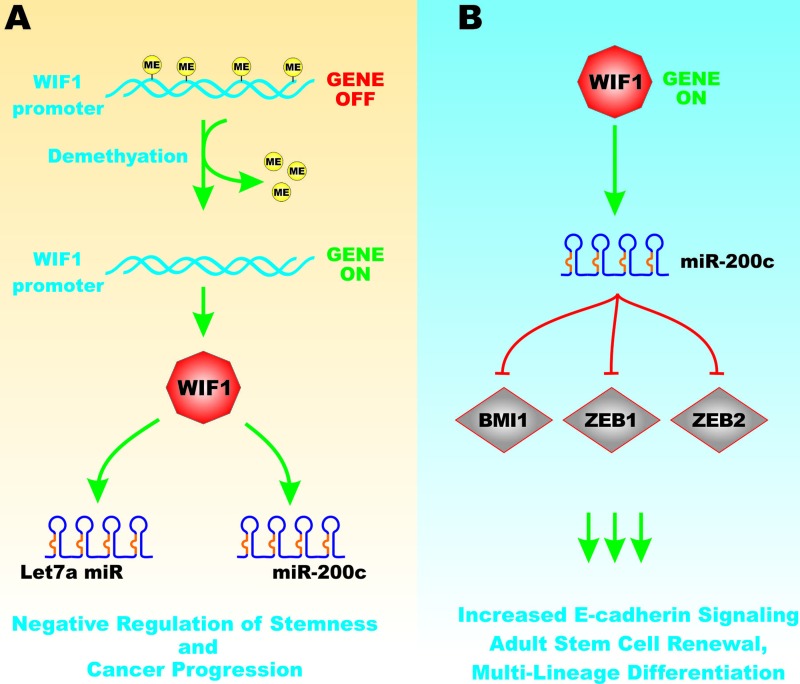
Effects of WIF-1 on mIRs and Genes involved in Cancer Progression Panel **A**. When the WIF1 promoter region is hypermethylated, the WIF1 gene is off. Upon demethylation of the WIF1 promoter region, the WIF1 gene is turned on and there is expression of WIF1 mRNA and protein. WIF1 serves to induce the expression of Let-7a and miR-200c that results in decreased expression of genes associated with pluripotency and stemness and results in more differentiation and cellular senescence. Panel **B**. When the WIF1 gene is on, it can induce miR-200C expression that can in turn effect the expression of genes involved in EMT such as BMI1, ZEB1 and ZEB2. This can result in increased E-cadherin signaling, adult stem cell renewal and influence multi-lineage differentiation. This figure is presented to provide the reader an idea of some of the mechanisms the WIF1 factor on genes involved in pluripotency, stemness and EMT and the roles that miRs may play in these processes.

The Wnt inhibitory protein-1 (WIF1) is a member of a family of proteins which bind Wnts and inhibit Wnt signaling. WIF1 is an extracellular protein which binds lipids and prevents Wnt-mediated signal transduction. WIF1 has been shown to decrease the quantity of salivary gland cancer stem cells and inhibit their anchorage-independent growth. Decreased expression of WIF1 was observed in salivary gland carcinoma ex-pleomorphic adenoma (CaExPA). WIF1 downregulation was observed to occur by WIF1 promoter hypermethylation and loss of heterozygosity (LOH). Re-expression of WIF1 resulted in a more differentiated phenotype and cellular senescence. This reduced the expression of pluripotency and stemness markers such as Oct-4 and c-Myc. In addition, WIF1 expression decreased Wnt-3A, TCF4, c-Kit and Myb expression that are associated with adult stem cell self renewal and multi-lineage differentiation [29]. The expression of the pri-miR-200c and pri-let7a were increased after restoration of WIF1 signaling. These miRs are proposed to be negative regulators of stemness and cancer progression. WIF1 has been shown to be a positive regulator of miR-200c. miR-200c decreased the expression of BMI1, ZEB1 and ZEB2 which resulted in an increase in E-cadherin expression [29]. Figure 7 presents a diagram of the effects of WIF1 and miRs on EMT and cancer progression.

## INVOLVEMENT OF TP53 AND MIRS IN REGULATION OF WNT/BETA-CATENIN PATHWAY AND EMT AND CSC GENERATION

**Figure 8:**
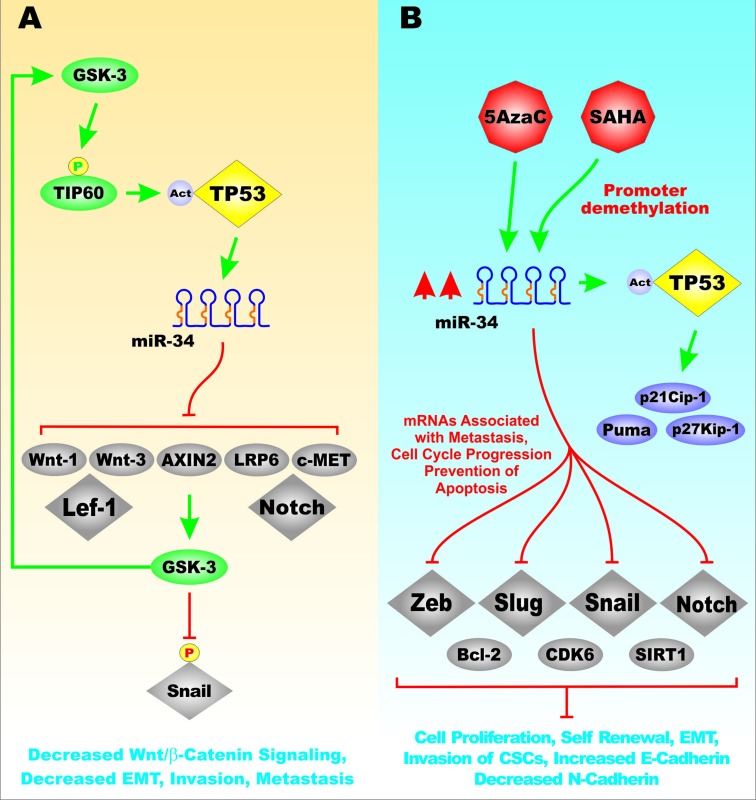
Effects of TP53 and miR-34 on the Wnt/beta-catenin Pathway Panel **A**. GSK-3 can phosphorylate and activate TIP60 which is a histone acetyl transferase. Activated TIP60 acetylates TP53 which results in its activation. Activation of TP53 can result in transcription of miR-34. miR-34 can suppress many genes in the Wnt/beta-signaling and other signaling pathways. One consequence of inhibition of Axin2 is that GSK-3 may be active. Active GSK-3 can phosphorylate Snail which leads to its inactivation. These events can lead to decreased Wnt/beta-catenin signaling, EMT, invasion and metastasis. Panel **B**. Effects of demethylation of the miR-34 promoter region by either 5AzaC or SAHA on miR-34 expression and the regulation of gene involved in cell proliferation, self-renewal, invasion, EMT, E-cadherin and N-cadherin expression. In addition, miR-34 expression can induce TP53 expression which can influence genes involved in cell cycle regulation and apoptosis such as p21^Cip-1^, p27^Kip-1^ and Puma. This figure is presented to provide the reader an idea of some of the mechanisms that TP53 and miR-34 play on regulation of genes involved in Wnt/beta-catenin signaling and EMT and how the TP53 protein may be regulated by GSK-3-induced TIP60 acetylation. Furthermore, the promoter region of the miR-34 gene is regulated by methylation that may be altered by 5AzaC or SAHA treatment.

TP53 transactivated miR-34, that in turn suppressed the transcriptional activity of TCF/LEF complexes by targeting the untranslated regions (UTRs) of genes regulated by Wnt/beta-catenin pathway [30, 31]. Loss of TP53 function was demonstrated to increase Wnt/beta-catenin signaling by loss of miR-34 interactions with target genes UTRs. miR-34 depletion suppressed TP53-mediated Wnt/beta-catenin target gene repression. Loss of p53 or miR-34 gene expression was demonstrated to contribute to neoplastic progression by triggering the Wnt/beta-catenin dependent, invasive activity of CRCs [30]. miR-34 was determined to regulate many genes involved in of Wnt/beta-catenin and EMT genes; namely *WNT1*, *WNT3*, *LRP6* (Low Density Lipoprotein Receptor-Related Protein 6), *AXIN2, CTNNB1* (beta-catenin), *LEF1* (Lymphoid enhancer-binding factor 1), *MET* (c-Met, aka hepatocyte growth factor receptor), *NOTCH1* (Neurogenic locus notch homolog protein 1) and *SNAI1* (Snail-1) resulting in suppression of TCF/LEF transcriptional activity and EMT. Loss of functional TP53 activity and miR-34 expression has been shown to alter the balance of CSCs and promote cancer progression [31]. Figure 8 presents an illustration of the effects of TP53 and miR-34 on the Wnt/beta catenin pathway, Snail expression and EMT.

**Figure 9:**
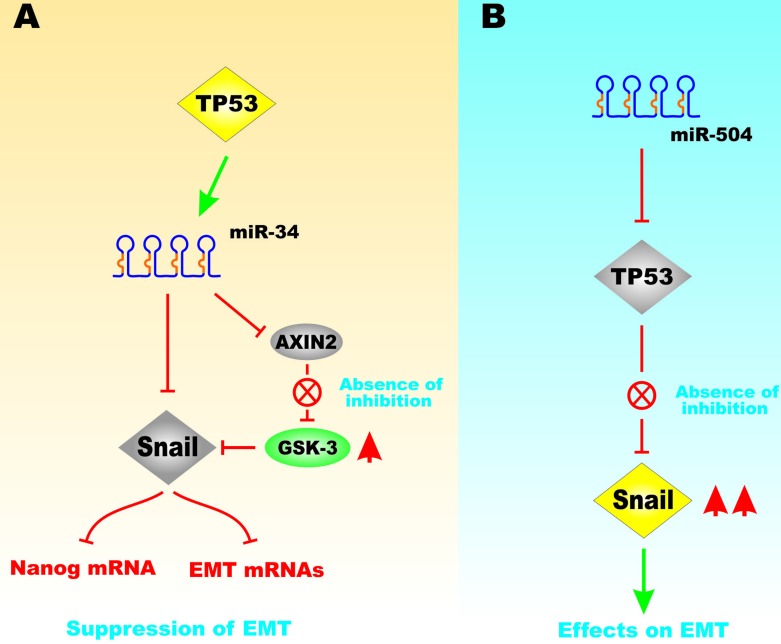
Effects of miR-34 and miR-504 on TP53-Regulated GSK-3 and Snail Expression Panel **A**. Effects of TP53 on miR-34 expression that can control the expression of Snail and Axin2 expression. When Axin2 is suppressed, GSK-3 can be activated that results in suppression of Snail and has negative effects on downstream Nanog and other mRNAs associated with EMT. Panel **B**. in contrast, if miR-504 is expressed, it can suppress TP53 expression the Snail is expressed that has positive effects on EMT. This figure is presented to provide the reader an idea of some of the mechanisms that the TP53 protein can regulate Snail, Axin2 and GSK-3 expression which will have effects on EMT.

miR-504 is a regulator of TP53. miR-504 binds two sites in the TP53 3′ untranslated region. Overexpression of miR-504 has been shown to suppress TP53 activity [32]. Obesity can induce breast tumor progression by increasing miR-504 that inhibits TP53. miR-504 has been shown to be induced by obesity and regulate TP53 in breast cancer [33]. Figure 9 presents a diagram of the effects of TP53, miR-34, miR504 on Snail expression and EMT.

miR-504 has also been shown to be important in brain cancer. Forkhead box protein P1 (FOXP1) is a target of miR-504 in glioma. Suppression of (FOXP1) by miR-504 resulted in decreased cell proliferation and promoted induction of apoptosis. In clinical samples, miR-504 was detected at lower levels [34].

In contrast, miR-504 was determined to be upregulated in nasopharyngeal carcinoma (NPC) radio-resistant cell lines. On the other hand, the expression of nuclear respiratory factor 1 (NRF1) and other proteins involved in mitochondrial metabolism was downregulated. These studies determined that miR-504 could target and decrease NRF1 expression. The expression of other proteins involved in mitochondrial respiratory function was also downregulated which led to impaired respiratory function. The levels of miR-504 were also determined in NPC patient sera after radiation and suggested that miR-504 may decrease NRF1 and be involved in radioresistance in NPC. It was suggested that targeting miR-504 might improve the radiation response [35].

In contrast, decreased expression of miR-504 was associated with a poor prognosis in high-grade glioma. The authors suggested that miR-504 levels could serve as an independent prognostic indicator for glioma. These studies have indicated that miR-504 may serve a tumor suppressor function in glioma. Other studies have proposed that miR-504 may serve as an oncogene by inhibiting TP53 expression [36].

The Trefoil factor 1 protein (TFF1 aka pS2) downregulates miR-504 expression, which can serve to downregulate TP53 expression. TFF1 is downregulated in human gastric cancer patients and genetic elimination in mice in results in gastric adenomas and carcinomas. TFF1 was determined to promote TP53 expression by inhibiting miR-504 expression [37].

Other studies have shown that miR-504 can inhibit the proliferation of hypopharyngeal squamous cell carcinoma (HSCC) by targeting CDK6. miR-504 expression was downregulated in HSCC patient samples while CDK6 expression was elevated. In HSCC cell lines, expression of miR-504 suppressed CDK6 expression while loss of miR-504 enhanced HSCC proliferation [38].

## MIR-129 REGULATION OF GSK-3 AND EMT

miR-129 has been shown to be important in various cancers including: breast, endometrial, and hepatocellular carcinoma [39–41]. miR-129 can regulate key genes involved in EMT such as Snail. Suppression of miR-129-5p is associated with a poor prognosis in breast cancer [41]. Silencing of miR-129-3b targets Aurora-A and promote EMT, invasion and metastasis in HCC [40]. miR-129 has been shown to regulate GSK-3beta [39]. Thus there are critical links between miR-129, GSK-3, EMT and other important components in cancer progression [39–41].

## GSK-3 EFFECTS ON CELL SURFACE ADHESION MOLECULES/RECEPTORS, EMT, CSCS AND INVASION

The *AXL* gene encodes a receptor tyrosine kinase that when aberrantly expressed is an oncogene. The role of Axl in esophageal squamous cell carcinoma (OSCC) has been recently investigated. Axl was determined to be overexpressed in tumor samples from patients with advanced stages of OSCC. Suppression of Axl inhibited proliferation, migration and invasion in animal studies. Suppression of Axl resulted in inhibition of NF-kappa-B induced gene expression and induction of GSK-3beta activity. This led to loss of mesenchymal proteins and increase in epithelial markers. The PI3K inhibitor Wortmannin was determined to inhibit NF-kappaB and to activate GSK-3beta signaling and suppressed OSCC proliferation. This suppression of OSCC proliferation was determined to be dependent on Akl. Thus, the NF-kappaB and GSK-3 pathways may have opposite roles in the development and progression of OSCC [42].

The miR-199-3p is involved in osteosarcoma. miR-199-3p inhibits the migration and invasion of osteosarcoma cells. Low levels of miR-199-3p are correlated with recurrence and lung metastasis in osteosarcoma patients. miR-199-3 is an independent predictor for poor prognosis. miR-199-3b was shown to suppress Axl expression and inhibit the progression of osteosarcoma. In osteosarcoma patient samples, an inverse relationship was observed between miR-199-3b and Axl expression [43]. The miR-34a also suppresses Axl expression in ovarian cancer. miR-34a expression was determined to be downregulated in ovarian cancer specimens [44].

miR-1, miR-133, miR-143 and miR-145 have been shown to effect the expression of genes involved in various signaling pathways important in cancer, mobility and adhesion such as TGF-beta, ErbB3, Wnt and VEGF. Ectopic expression of miR-1 or miR-145 suppressed viability and colony formation of a gallbladder cancer cell line, as well as Axl and VEGF-A expression [45]. Axl has also been shown to be involved in acquired resistance to gelfitinib and erlotinib in NSCLC. Axl can regulate the expression of miR-374a and miR-548b and target Wnt5a and cyclin B1 (*CCNB1*) in gefitinib-resistant lung cancer cells. A poor disease-free survival was associated in NSCLC patients that had high expression of Axl and miR-374a [46]. miR-34a and TP53 have been shown to regulate Axl expression in B-cell chronic lymphocytic leukemia. This tumor-suppressor network has implications for-TP53 negative patients [47].

A head and neck cancer (HNC) cell line (HN5-ER) was developed from HN5 cells which displayed an EMT phenotype and eroltinib-resistance. The HN5-ER cells displayed reduced expression of miR-34a. Upon increased miR-34a expression in these cells, Axl expression decreased as well as erlotinib-resistance. Upon analysis of 302 HNC patient samples, it was determined that high Axl expression was associated with poorer survival [48].

Inverse Axl and miR-34a expression has also been observed in breast cancer cell lines and tumor samples [49]. In another study, an inverse expression of miR-34a and Axl mRNA expression was observed in breast, CRC and NSCLC cell lines. miR-199a/b may also regulate Axl expression. However, miR-199a/b expression was determined to be suppressed in all the cancer cell lines examined in this study. In cells where the miR-34a promoter was methylated, there was less expression of miR-34a while the miR-199a/b was completely methylated in all samples analyzed. In 44 NSCLC tissue samples, miR-34a and miR-199a/b were determined to be downregulated. Interestingly, longer patient survival was correlated with higher levels of miR-34a expression [50].

GSK-3beta has been shown to interact with focal adhesion kinase (FAK), Ras-related C3 botulinum toxin substrate 1 (Rac1) and c-Jun-N-terminal kinase (JNK) in glioblastoma. These interactions are important in invasion. Suppression of GSK-3beta was determined to inhibit invasion both *in vitro* and in mouse tumor models of invasion. GSK-3beta inhibition suppressed FAK, JNK and Rac1 and resulted in decreased lamellipodia and invadopodium-like structures. This resulted in changes in the localization and activity of Rac1 and F-actin. In addition, decreased levels of matrix metallo-proteinases were observed. These results point to the potential use of GSK-3 beta inhibitors in the treatment of glioblastomas [51].

The chemokine (C-X-C Motif) Receptor 2/chemokine (C-X-C Motif) Ligand 5 (CXCR2/CXCL5) interaction have been shown to regulate the PI3K/PTEN/Akt/mTORC1/GSK-3-beta/Snail pathway in HCC. This pathway can influence EMT. High levels of CXCR2 were associated with poor prognosis in HCC patients. The PI3K/PTEN/Akt/mTORC1/GSK-3-beta/Snail pathway and EMT phenotype in HCC cells was associated with high levels of CXCR2 and CXCL5 expression [52].

The twist-related protein 1 (TWIST1) has been shown to be involved in oral tongue squamous cell carcinoma (OTSSC) invasion. Elevated TWIST1 was associated with a poor prognosis in OTSSC patients. TWIST overexpression in OTSCC lines was associated with phosphorylation of GSK-3beta at S9 [53].

**Figure 10:**
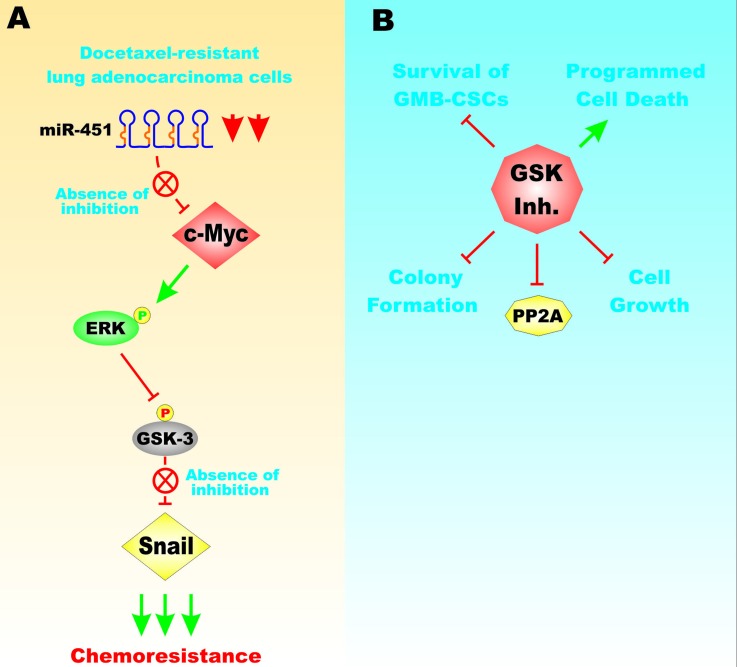
Effects of GSK-3 on EMT and Drug Resistance Panel **A**. miR-451 can suppress c-Myc expression and miR-451 is down-regulated in docetaxel-resistant lung adenocarcinoma cells. c-Myc overexpression can induce ERK activation which in turn results in GSK-3 phosphorylation and its inactivation. This can result in Snail stabilization which in turn leads to chemoresistance. Panel **B.** GSK-3 is involved in the survival of GMB-CSCs. Suppression of GSK-3 with GSK-3 inhibitors can result in inhibition of PP2A activity, cell growth, colony formation and induced programmed cell growth. This figure is presented to provide the reader an idea of some of the mechanisms that GSK-3, c-Myc and miR-451 can be involved in cell growth and chemoresistance.

A pathway consisting of miR-451, c-Myc, ERK, GSK-3beta and Snail has been shown to be important in the docetaxel-resistance of lung adenocarcinoma cell models (LAD) which have some phenotypes associated with cells having undergone EMT and have increased invasive and migratory properties. c-Myc was shown to be a target of miR-451. Figure 10, Panel A presents an illustration of the effects of miR-451 on c-Myc expression and chemoresistance. Overexpression of c-Myc could induce the ERK-dependent phosphorylation of GSK-3 which resulted in its inactivation and induction of Snail. miR-451 normally suppresses the EMT phenotype. miR-451 was down regulated in docetaxel-resistant LAD cells [54].

GSK-3beta may be important in regulating the survival in GBM cells with CSC-like properties. Inhibiting GSK-3 activity suppressed proliferation, colony formation and induced programmed cell death. GSK-3beta had effects on protein phosphatase 2A (PP2A) and suppression of GSK-3beta resulted in decreased levels of PP2A. GSK-3beta may be important in glioblastoma CSC-like cells [55]. Figure 10, Panel B presents an overview of the effects of inhibiting GSK-3 in glioma CSCs.

These models presented in Figure 10 document the different effects that GSK-3 can have on cell proliferation and drug resistance. In Panel A) the effects of miR-451 on drug resistance and the involvement of c-Myc, GSK-3 and Snail expression is presented and GSK-3 is associated with the survival of GMB-CSCs. In Panel B, suppression of GSK-3 is associated with inhibition of cell growth.

Protocadherin 9 (PCDH9) has been determined to be either lost or down-regulated in HCC. PCDH9 is involved in tumor migration and EMT. PCDH9 has been shown to increase the activity of GSK-3beta by inhibiting Snail. Thus, PCDH9 could be a novel target to prevent metastasis in HCC [56].

**Figure 11:**
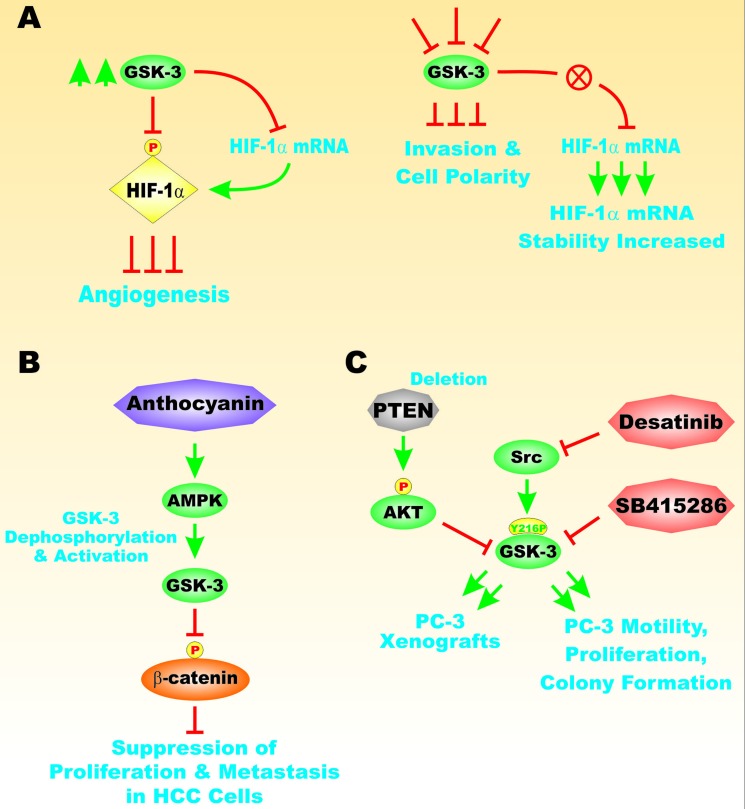
Effects of GSK3 on HIF-1alpha, Motility and Metastasis Panel **A**. GSK-3 can result in phosphorylation HIF-1alpha that results in its inactivation. Suppression GSK-3 can result in HIF-1alpha mRNA stabilization and inhibition of invasion and cell polarity. Panel **B**. Anthocyanins can activate 5′AMP protein kinase (AMPK) that can in turn lead to GSK-3 activation and suppression of beta-catenin. Panel **C**. Effects of suppression of PTEN on Akt activation and inhibition of GSK-3 which can lead to PC3 xenograft formation upon injection with PTEN-deficient PC3 cells. Src can phosphorylate GSK-3 on Y216 which result in GSK-3 activation. Inhibition of Src or GSK-3 with Desatinib or SB415286 respectively, can have effects on PC3 xenograft formation, PC3 motility, cell proliferation and colony formation. This can lead to suppression of proliferation and metastasis of HCC cells. This figure is presented to provide the reader an idea some of the mechanisms that GSK-3, can be involved in angiogenesis, tumor formation and metastasis.

Epidermal growth factor (EGF) has been shown to promote the stability of Snail by suppressing GSK-3beta activity in prostate cancer. Inhibition of GSK-3beta results in Snail transcription [57]. In another study, Akt has been shown to be important in the regulation of GSK-3beta and Snail after basic fibroblast growth factor (bFGF) stimulated induction of EMT in prostate cancer [58]. Figure 11 presents an overview of some of the mechanisms which can alter GSK-3 activity and invasion of cancer cells.

GSK-3beta has been shown to be involved in the regulation of HIF-1alpha expression Suppression of GSK-3beta resulted in increased HIF-1alpha expression in osteocarcoma cells by increasing the stability of HIF-1 alpha mRNA [59]. Figure 11, Panel A, presents a diagrapm of the interactions between GSK-3 and HIF-1alpha and their importance in events involved in cancer progression.

Cell polarity is important for cell migration. GSK-3 has been shown to be important in cell polarity. S9-phosphorylated GSK-3beta was determined to be critical for glioma cell invasion. Downregulation of GSK-3alpha and GSK-3beta suppressed glioma invasion, while EGF was shown to regulate both GSK-3alpha and GSK-3beta. It was determined that only GSK-3beta phosphorylated at S9 was enriched at the glioma cell's leading edge in migration assays. EGF was determined to upregulate GSK-3beta through atypical PKC pathways. In contrast, GSK-3alpha was not upregulated in a similar fashion. These studies point to the importance of GSK-3beta in glioma cell invasion and polarity as well as point to some of the different effects of GSK-3alpha and GSK-3beta [60].

Links between adenosine monophosphate kinase (AMPK) and GSK-3 in the regulation of metastasis and proliferation have been described in HCC cells exposed to anthocyanins obtained from the Korean wild berry Meoru. Hep3B cells treated with anthrocyanins suppressed beta-catenin activation by GSK-3beta. Figure 11, Panel B presents an illustration of the effects of anthocyanins on GSK-3 activity and the beta-catenin pathway. The dephosphorylation of GSK-3beta (activation) was determined to be dependent on AMPK activation [61].

The levels of tuberous sclerosis complex 2 (TSC2, aka tuberin) and GSK-3beta were examined in clinical HCC patient samples as well as precancerious tissues and normal livers. GSK-3beta and TSC-2 were detected at lower levels than in in normal liver and precancerous controls [62].

Maelstrom (MAEL) is a novel oncogene located at 1q24 which is frequently amplified in HCC. MAEL was shown to increase Akt activity which resulted in GSK-3 phosphorylation and Snail stabilization. This led to EMT, tumor invasion and metastasis. MAEL was also shown to increase the expression of stemness and multidrug resistance genes as well as CSC markers at least at the mRNA level [63].

An Akt-resistant pathway for GSK-3 activation has been described in prostate cancer cells. GSK-3beta activity has been associated with both tumor promoter and tumor suppressor activities. Prostate cancers often display elevated levels of Akt. Src may be responsible for GSK-3 phosphorylation at Y216 which can result in activation of GSK-3 even in the presence of activated Akt. Figure 11, Panel C, presents an illustration of the importance of GSK-3 in prostate cancer growth and progression. Suppression of GSK-3 by treatment with the GSK-3 inhibitor SB415286 suppressed motility, proliferation and colony formation in PC3 prostate cancer cells which express large amounts of activated Akt due to PTEN deletion. Desatinib treatment inhibited proliferation and invasion as well as impaired PC3 xenograft formation [64].

**Figure 12:**
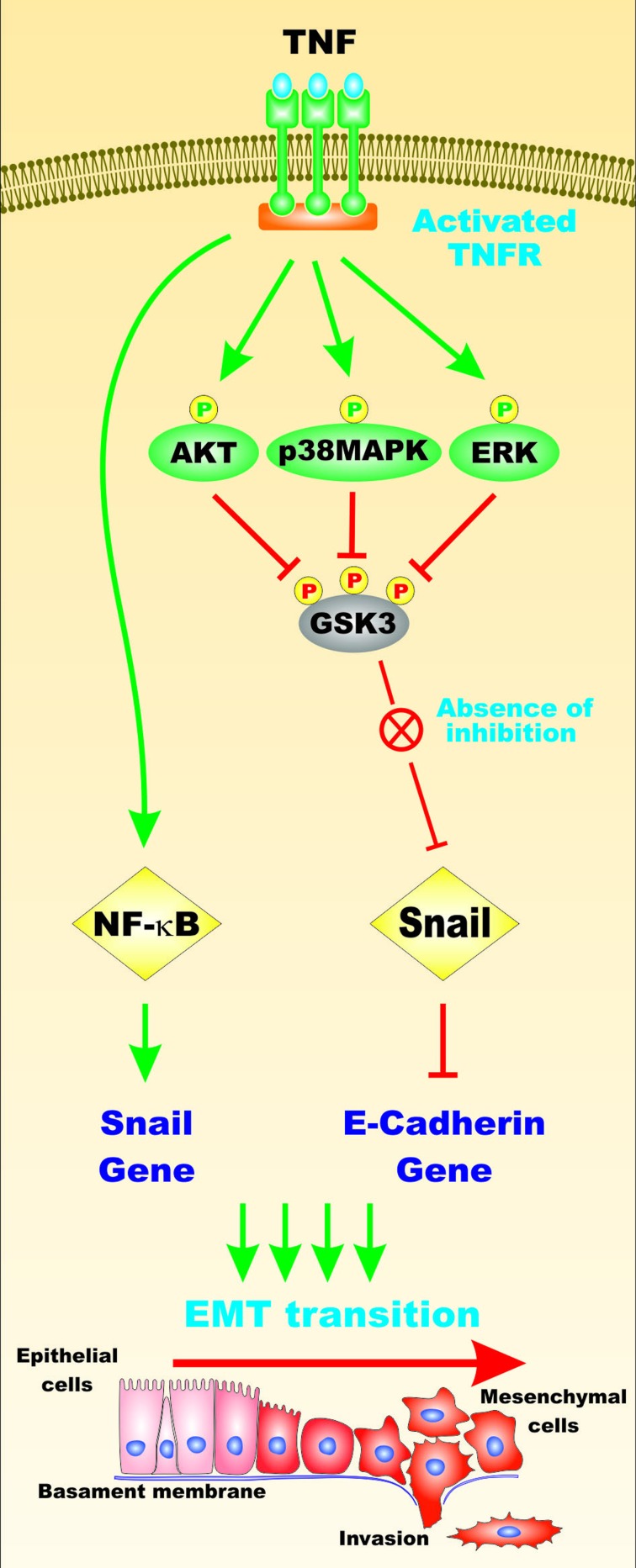
Effects of TNF on GSK-3, NF-kappaB and Snail Activities and EMT TNF can activate multiple signaling pathways including: Akt, p38MAPK and ERK that can phosphorylate and inactivate GSK-3 that can promote Snail activity. Snail can prevent the expression of the E-cadherin gene and contribute to EMT. TNF can also activate NF-kappaB which can stimulate Snail gene expression which also promotes EMT. This figure is presented to provide the reader an idea of some of the mechanisms that TNF-alpha can regulate GSK-3, Snail and NF-kappa-activity and EMT.

The Akt/GSK-3beta pathway is required for TNF-alpha induced Snail stabilization and EMT in prostate cancer PC3 cells. In these studies, TNF-alpha was shown to induce NF-kappaB, Akt, ERK and p38^MAPK^ and activated Akt and inhibited GSK-3beta activity which resulted in elevated Snail activity [65]. The effects of TNF-alpha on GSK-3 and Snails activities is depicted in Figure 12.

Slug is a zing finger-containing transcriptional repressor which is regulated by GSK-3. It suppresses E-cadherin expression and stimulates EMT. In NSCLC, it was demonstrated that the expression of Slug was associated with the levels of S9-phosphorylated GSK-3beta. GSK-3 can phosphorylate Slug which results in protein turnover. Another protein important in regulation of Slug is the carboxyl terminus of Hsc70-interacting protein (CHIP) which interacts with Slug. Suppression of CHIP was shown to stabilize Slug and inhibited its ubiquitination and degradation. Increased levels of Slug may lead to reduced E-cadherin expression and promote migration, invasion and metastasis [66].

The enhancer of zeste homolog 2 (EZH2) is important in tumor development and progression EZH2 may also be a substrate for GSK-3. The role of GSK-3beta and EZH2 has been investigated in nasopharyngeal carcinoma (NPC). EZH2 levels were determined to correlate with S9-phosphorylated GSK-3beta in NPC patient samples. Inhibition of GSK-3beta in NPC cell lines lead to upregulation of EZH2. This resulted in increased local invasiveness which was shown to be dependent on EZH2 [67].

The role of the *FAT10* oncogene has been investigated in HCC. The *FAT10* gene is located at 6q21.3. 6q21.3 is frequently amplified in HCC. FAT10 is also known as ubiquitin D. It was determined recently that expression of FAT10 correlated with poor prognosis and reoccurrence. FAT10 overexpression stimulated proliferation and prevented apoptosis. Suppression of FAT10 reversed these properties. FAT10 induced EMT and invasion. Suppression of Akt inhibited the proliferative and anti-apoptotic effects of FAT10 on the HCC cells [68].

Fas signaling can induce EMT in gastrointestinal (GI) tumors. Fas-ligand (FasL) was shown to suppress E-cadherin transcription by increasing Snail expression. FasL induced ERK activity which resulted in GSK-3beta phosphorylation at S9. Snail then was able to associate with beta-catenin in the nucleus which enhanced its transcriptional effects. The expression of these proteins was examined in human GI cancer specimens. Enhanced levels of FasL, Snail, beta-catenin and S9-phosphorylated GSK-3beta were detected in GI cancer progression [69].

The Klotho protein is a beta-glucuronidase which can hydrolyze steroid beta-glucuronides. Klotho is important in diabetes and aging. Recently the Klotho protein has been proposed to be a tumor suppressor protein in many cancer types. Klotho is expressed in renal tubular epithelial cells and appears to function as an anti-apoptotic protein. The role of Klotho in renal cell carcinoma (RCC) has been investigated. Klotho levels were correlated negatively with tumor size in 125 RCC patient samples. Overall patient survival was correlated with high Klotho expression. Klotho appeared to inhibit the PI3K/PTEN/Akt/GSK-3beta/Snail pathway. Low Klotho levels were associated with Snail and activated Akt-1 expression. The authors proposed that Klotho suppresses PI3K/PTEN/Akt/GSK-3/Snail signaling, EMT and invasion. Thus Klotho might be a useful therapeutic target in patients with advanced RCC [70].

TNF-alpha can induce EMT in the CRC line HCT-116. This results in invasion and metastasis. EMT was characterized by increased expression of N-cadherin and fibronectin and decreased E-cadherin and Zona occludin-1 expression. In these CRC cells, increased Snail but not Slug, Zeb or Twist expression was detected. Snail was shown to be important for EMT in HCT-116 cells. The effects of TNF-alpha on Snail activity were determined to be mediated by Akt activation and subsequent GSK-3beta expression. These studies provide information concerning the role of inflammation and CRC metastasis [71].

The role of GSK-3beta in invasiveness of pancreatic cancer has been investigated. Inhibition of GSK-3beta suppressed the proliferation of pancreatic cancer cells and sensitized them to gemcitabine and ionizing radiation. Decreases in the levels of cyclin D1 expression and Rb phosphorylation were observed. Suppression of GSK-3beta also changed the location of Rac-1 and F-actin and altered lamellipodia microarchitecture. Decreased levels of MMP2 and FAK phosphorylation were also observed [72].

The Raf kinase inhibitor protein (RKIP) has been shown to bind GSK-3beta and maintain the protein in its active configuration. Removal of RKIP stimulates activation of p38^MAPK^ which phosphorylates GSK-3beta on T390 and inhibits its activity. This relieves the GSK-3beta- mediated inhibition of cyclin D, beta-catenin and Slug which promotes EMT. RKIP levels in CRC have been shown to correlate with GSK-3beta. The authors have suggested that RKIP/GSK-3 maybe be a promising therapeutic target [73]

Galectin-3 (Gal-3) is a member of the beta-galactoside-binding protein. It may be involved in the regulation of pancreatic cell mobility. Silencing Gal-3 suppressed cell migration and invasion but not proliferation [74].

Sirtuin2 (SIRT2) is a NAD-dependent protein deacetylase. The sirtuins are involved in a number of key biochemical processes including telomere maintenance. SIRT2 has been shown to be important in the motility and invasiveness of HCC. Suppression of SIRT2 prevented EMT phenotypes. SIRT2 was shown to control the deacetylation and activity of Akt that in turn regulated GSK-3beta/beta-catenin and EMT [75].

GSK-3beta can phosphorylate the long form of the collapsin response mediator protein-1 (LCRMP-1), a protein that is important in migration and invasion. LCRMP-1 is associated with a poor clinical outcome in non-small cell lung cancer (NSCLC) patients. GSK-3beta can phosphorylate LCRMP-1 on T268. This phosphorylation event is important in the stimulation of filopodia, migration and invasion. Patients with low GSK-3beta S9 phosphorylation and high LCRMP-1 were determined to have a worse overall survival than patients with high GSK-3beta S9 and low LCRMP-1 [76].

Akt regulates GSK-3beta which in turn controls ZEB1 transcription. ZEB1 modifies the expression of cytokeratins, vimentin and MMP9. Zeb1 expression was shown to be essential for invasion in bladder cancers as well as metastasis *in vivo* [77].

Inhibition of GSK-3 suppressed the motility of melanoma cells. Inhibition of GSK-3 suppressed N-cadherin and Slug expression. GSK-3 inhibitors also blocked FAK phosphorylation. These studies describe novel roles for GSK-3 in controlling the motility of melanoma cells by regulation of N-cadherin and FAK expression [78].

The anti-tumorigenic cytokine TNF-alpha can inhibit GSK-3beta activity by stimulating Akt to phosphorylate GSK-3beta at S9 in renal carcinoma cells (RCC). Treatment of RCC cells with lithium resulted in suppression of GSK-3beta and increased levels of MMP9 and the induction of EMT. Introduction of an activated *GSK3B* gene into RCC cells inhibited TNF-alpha-induced anchorage-independent growth as well as tumorigenicity in mice. In contrast, introduction of a kinase-deficient *GSK3B* gene into RCC cells promoted EMT, anchorage-independent growth and tumorigenicity. Thus GSK-3beta plays a key role in TNF-alpha induced tumorigenesis of RCC [79].

Introduction of a kinase-dead (KD) *GSK3B* construct into estrogen receptor positive MCF-7 breast cancer cells increased their resistance to the chemotherapeutic drug doxorubicin and the estrogen receptor antagonist 4-hydroxytamoxifen (4HT) [80]. Furthermore, introduction of an activated GSK-3beta construct increased the sensitivity of the MCF-7 cells to doxorubicin and 4HT. The GSK-3beta KD also increased the colony formation of the cells in soft agar in the presence of doxorubicin.

Rhubarb can activate GSK-3beta and inhibit HCC metastasis by enhancing the degradation and nuclear translocation of beta-catenin. Rhubarb also downregulated cyclin D, T-box transcription factor-3 (Tbx3), c-Myc, MMP9 and contactin-1. Rhubarb suppressed GSK-3beta phosphorylation at S9 and disrupted Wnt signaling [81].

Migfilin is a component of focal adhesion complexes. Migfilin is thought to be involved in cell-extracellular matrix adhesion and control of motility in ESCCs. Migfilin was shown to be associated negatively with metastasis and migfilin inhibited cell motility by decreasing the level of free beta-catenin. This occurred in part by migfilin promoting the association of GSK-3 and beta-catenin [82].

Leptin is an important hormone made by adipose cells and is important in regulating hunger and diabetes. Leptin has been shown to induce Akt to phosphorylate GSK-3beta. This results in a decrease in formation of the GSK-3beta/LKB1/Axin complex which results in upregulation of beta-catenin. Leptin also induces the expression of metastasis-associated protein (MTA1) which is important in Wnt signaling. Silencing of MTA1 was shown to prevent leptin-induced Wnt1 expression. In leptin-treated breast tumors, a cross-talk between leptin and MTA1/Wnt signaling was observed that may be important in EMT [83].

## EFFECTS OF THE PI3K/PTEN/AKT/MTORC1/GSK-3 PATHWAY ON INVASION AND CSCS

The PI3K/PTEN/Akt/mTORC1 pathway has been shown to be important in the invasive and migratory properties of certain cancers including ovarian cancer [84]. Upregulation of the IGF-1R/mTOR and mevalonate-isoprenoid biosynthesis (MIB) pathways has been observed in colorectal cancer cells with CSC properties. Inhibition of IGF-1R/mTOR suppressed CRC CSC growth and the expression of CSC markers [85].

Greater than 80 proteins associated with protein synthesis have been observed to be differentially expressed in CSCs prepared from breast CSCs in comparison to their adherent breast cancer cells that they were derived from. Rapamycin was shown to be very potent in inhibiting mammosphere formation. Methionine restriction, which mimics caloric restriction, was also determined to inhibit CSCs preferentially [86].

Combining the antibiotic salinomyin (SAL), which may function as a potassium ionophore, with metformin was shown to have synergistic effects on the induction of cell death in NSCLC CSCs. This occurred in NSCLC CSCs regardless of their EGFR, KRas, echinoderm microtubule associated protein like 4/anaplastic lymphoma receptor tyrosine kinase (EML4/ALK) and serine/threonine kinase 11/liver kinase B1 (STK11/LKB1) status. Higher levels of inhibition of downstream targets of EGFR (Akt, ERK1,2) and mTOR (p70S6K) were observed when metformin and SAL were combined together than after single agent addition [87].

## WNT PATHWAY ASSOCIATED PROTEINS AND CSCS

The B cell-specific Moloney murine leukemia virus integration site 1 (BMI1) protein (aka polycomb group RING finger protein 4) has been shown to activate the Wnt pathway by suppressing the DKK family of Wnt inhibitors. The repression of DDK1 by BMI1 resulted in increased expression of the Wnt target c-Myc. This in turn led to the transcriptional activation of BMI1. These studies elucidated a positive feedback BMI1 loop which may be important in maintaining the CSC phenotype [88].

Another group of proteins which suppresses Wnt signaling are the DDK family of Wnt inhibitors. Overexpression of certain DKKs in the hematopoietic niche can result in suppression of HSC long-term repopulating activity [89]. Forced overexpression of DKK1 in osteoblasts leads to osteopenia that can disrupt the HSC niche as well as the function of HSCs. DKK1 also plays important roles in inhibiting fracture repair. DKK activation in osteoblasts can lead to certain forms of osteoporosis. DKK1 has been implicated in the progression and metastases of many diseases including multiple myeloma (MM), breast cancer, and in those prostate cancers where there is bone pathology [90]. Osteolytic bone lesions and bone fractures can occur in MM patients who express DDK1 [90]. Other cancers also expressed decreased levels of DDKs. Lower levels of DDK4 were detected in HCC [92]. Certain DKK genes were determined to be epigenetically inactivated in gastrointestinal and CRC cancers [92].

Downregulation of CD133 reduced the metastasis of melanoma cells to the spinal cord. The expression of genes encoding Wnt inhibitors was observed to be upregulated when CD133 (Prominin-1 a cell surface molecule often associated with CSCs) was down regulated in melanoma cells [93].

## REGULATION OF FZD PROTEINS BY MIRS AND TP53

**Figure 13:**
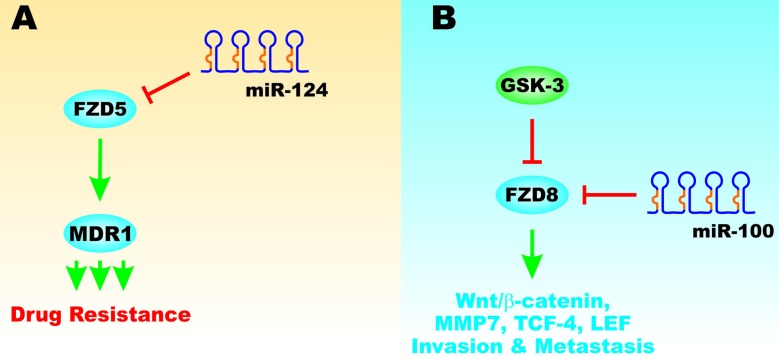
Effects of miRs and FZD on Drug Resistance, Invasion and Metastasis Panel **A**. The FZD5 gene can influence the expression of the MDR1 gene which can result in chemotherapeutic drug resistance. Panel **B**. GSK-3 can suppress FZD8 which has effects on Wnt/beta-catenin signaling, invasion and metastasis. miR-100 can also suppress FZD8 expression which has effects on Wnt/beta-catenin signaling, invasion and metastasis. This figure is presented to provide the reader an idea of some of the mechanisms that FZD can regulate proteins involved in drug resistance and how they can be regulated by miRs.

The FZD proteins play essential roles in Wnt-mediated signaling pathways. FZD mRNAs are regulated by diverse miRs. FZD5 can be involved in the regulation of the multidrug resistance gene (MDR1, ABCB1, P-glycoprotein) (Figure 13, Panel A). FZD5 can in turn be regulated by miR-124 [94]. miR-124 expression can block drug resistance (Figure 13, Panel B). GSK-3beta expression can block FZD8 expression which is important in Wnt/beta-catenin signaling, invasion and metastasis. miR-100 can inhibit FZD8 expression that has effect on breast cancer invasion and metastasis (Figure 13, Panel B) [95]. The miR-199a-5b regulates FZD6 in CRC cells. FZD6 is detected at elevated levels in CRC tumor samples as compared to adjacent non-cancerous tissue [96].

**Figure 14:**
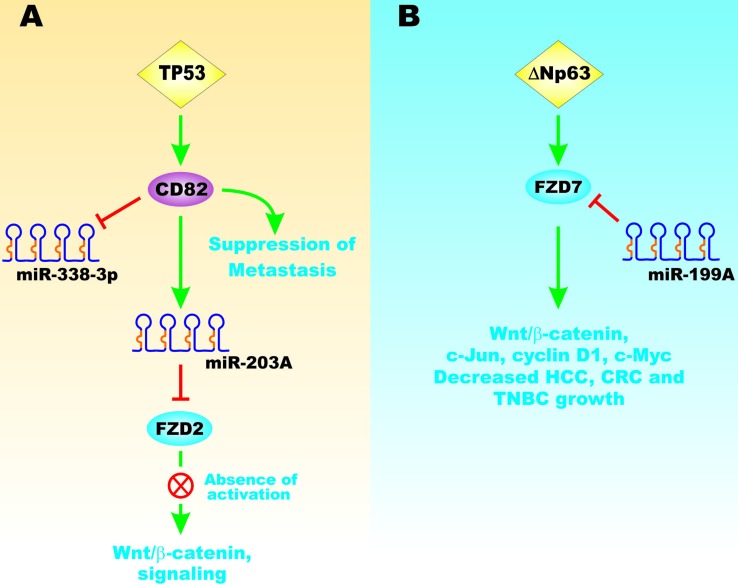
Effects on TP53 and ∆Np63 on FZD Gene Expression and Wnt/beta-catenin Signaling Panel **A**. TP53 can induce the CD82 gene that can suppress metastasis. CD82 can also induce expression of miR-203A and inhibit the expression of miR-338-3p. miR-203A can suppress FZD2 expression which in turn resulted in inhibition Wnt/beta-catenin signaling. Panel **B**. ∆Np63 can result in FZD7 expression that can have effects on Wnt/beta-catenin signaling and is associated with a poor prognosis in TNBC. miR-199A can inhibit FZD7 expression which in turn inhibits Wnt/beta-catenin expression. This figure is presented to provide the reader an idea of some of the mechanisms that TP53 and ∆Np63 can regulate FZD expression that affects EMT.

TP53 can also regulate the cluster of differentiation 82 (*CD82*) gene that encodes a membrane glycoprotein. CD82 is important in the suppression of metastasis. CD82 can also modulate miR-203 positively and miR-338-3p negatively. miR-203 can inhibit FZD2 mRNA and block Wnt/beta-catenin signaling (Figure 14, Panel A) [97].

The TP53 related gene ∆Np63 can regulate the expression of FZD7 (Figure 14, Panel B). FZD7 is also involved in regulating Wnt/beta-catenin signaling. Overexpression of FZD7 is associated with a poor prognosis in triple negative breast cancer (TNBC). miR-199A can suppress FZD8 mRNA which inhibits Wnt/beta-catenin signaling [98].

## WNT SIGNALING IN STEM CELL SELF-RENEWAL

Stem cells have the capacity to self-renew as well as produce specialized cells. The choice to self-renew or produce specialized cells may be dictated by extrinsic signaling factors which are present in the niche or in an area which functions as a signaling center. Many of these factors act in the near vicinity of the stem cells in order to tightly regulate them. Key pathways involved in regulating the niche are: Wnt, bone morphogenic protein (BMP), Hh and Notch [99–102]. Altering the activity of the Wnt pathway has been shown to affect HSC. Overexpression of Axin decreased the number of transplantable HSCs [103]. Activation of Wnt signaling by introduction of mutant forms of beta-catenin can increase hair follicle morphogenesis and hair tumors [104]. Treatment of HSC with the Wnt-3a protein increased self-renewal and long term reconstitution in irradiated mice [100]. Wnt proteins have also been shown to promote maintenance of pluripotency in murine ESCs [105, 106]. The mechanisms by which Wnt signals maintain stemness are not fully elucidated. Clevers and Nusse [99] have suggested that stem cells are intrinsically destined to differentiate and that Wnt signals could block the differentiation step by suppressing differentiation-specific genes as Wnt signals can also suppress the expression of certain genes [99].

## THE HEDGEHOG (HH) PATHWAY AND CSCS

Crocetinic acid can be purified from the commercial saffron compound crocetin. Crocetinic acid inhibited the expression of both the sonic hedgehog ligand (Shh) and smoothened (Smo) in pancreatic CSCs. Smo is a G protein-coupled receptor and is a frizzled class receptor. Crocetinic acid inhibited the number and size of pancreatic CSCs [107].

The Hh pathway may be responsible for the stemness properties of certain breast cancer cells after exposure to chemotherapeutic drugs such as docetaxel. Treatment of breast cancer cells with docetaxel resulted in release of Shh and increased expression and nuclear translocation of Gli-1. These effects appeared to have been more prominent in the breast CSCs than in the breast cancer cells lacking the CSC properties (aka., the bulk cancer). The authors concluded that Hh pathway activation by docetaxel did not induce chemosensitivity but instead may be required for survival and expansion of the breast CSCs after chemotherapy [108].

Curcumin may influence CRC CSC. Curcumin may effect multiple signaling pathways including Wnt/beta-catenin, Shh, Notch and PI3K/PTEN/Akt/mTORC. In addition, curcumin may have effects on mIRs and EMT [109].

Mammospheres prepared from the breast cancer cell line MCF-7 cells have been shown to be sensitive to the bacteria gram positive antibiotic salinomycin, but resistant to the chemotherapeutic drug paclitaxel. This is in contrast to the parental bulk cancer MCF-7 cells. PTCH, SMO, Gli1 and Gli2 were upregulated in the MCF-7 mammospheres and their expression was sensitive to salinomycin but not paclitaxel. Salinomycin inhibited the expression of gene targets of the Hh pathway including: c-Myc, Bcl-2 and Snail. The expression of SMO and Gli1 was correlated with the CD44+/CD24- phenotype which is often associated with breast CSCs. These characteristics were linked with a shorter overall and disease free survival of breast cancer patients being treated with chemotherapy [110].

Gastric CSCs which express CD44(+)/Musashi-1(+) were determined to express high levels of Shh and Gli1. Suppression of Gli1 activity inhibited the doxorubicin-resistance of the cells [111].

Gli1 has been shown to be a regulator of the Sox2 transcription factor in NSCLC. Suppression of Hh or Gli1 inhibited the self-renewal of NSCLC CSC. In addition, suppression of EGFR and Hh was shown to reduce the viability of NSCLC and the self-renewal of NSCLC CSCs [112].

The Hh pathway is important in colon carcinoma EMT and CSCs [113, 114]. Colon carcinomas are derived from the intestinal epithelium. The intestinal epithelium undergoes constant renewal by the progeny of stem cells which reside in the bottom of the crypts of Lieberkuhn. Normal stem renewal is regulated by Wnt/TCF signaling. Greater than 90% of colon carcinomas contain inactivating mutations in the *APC* gene or activating mutations in the beta-catenin gene (*CTNNB1*). These mutations result in abnormal activity of the TCF family of transcription factors which contribute to the genesis of colon carcinomas. The abnormal activity of Wnt/TCF has been thought to drive the expansion of colon carcinoma CSCs and tumor development [115, 116].

An intriguing interrelationship between Wnt/TCF and Hh/GLI signaling pathways has been documented in the metastatic transition of colon carcinomas [113]. Namely, the activity of the Wnt/TCF pathway decreased while the activity of the Hh/Gli pathway increased during EMT. The Hh/Gli pathway drove the downregulation of Wnt/TCF pathway which resulted in the enhancement of more primitive embryonic stem cells. Interestingly, this group observed that TCF blockade did not suppress tumor growth but importantly, it increased Hh/Gli signaling. Enhanced Gli1 levels were determined to drive the transition of colon carcinomas to a reprogrammed, embryonic stem cell-like, metastatic state and the Wnt/TCF pathway was suppressed. This switch was identified in fresh tumor samples from patients. Furthermore, increased expression of Wnt pathway inhibitors such as DKK1 and sFRP1 in metastatic colon carcinomas and liver metastases was observed. The authors suggested that the phenotype of advanced and metastatic colon carcinomas was only maintained in xenografts. In contrast, in *in vitro* studies with non-metastatic colon carcinomas, both the Wnt/TCF and Hh/Gli pathways were expressed and necessary. Wnt/TCF activity antagonized tumor and metastatic growth and inhibition of Wnt/TCF promoted enhanced metastatic growth. Thus, targeting Hh/Gli may be more important than suppressing the Wnt/TCF pathway in certain cancers.

This group also observed that when colon carcinomas with non-metastatic properties and colon carcinomas with metastatic properties were cultured *in vitro*, the Wnt/TCF and Hh/Gli pathways interacted at multiple levels due to the tissue culture microenvironment. Both pathways may regulate c-Myc expression while the Wnt/TCF pathway may suppress Gli activity. The authors demonstrated that Wnt3/beta-catenin regulated Gli3 transcription resulting in Gli3 being a repressor of Gli1. In contrast, when Hh/Gli1 was enhanced, Gli3 was repressed. Beta-catenin can negatively regulate Gli3 and positively regulate Gli1 independently of TCF in colon carcinoma CD133+ stem cell populations [114].

CRC CSC stem cell populations are clearly important in CRC carcinogenesis [115, 116]. The effects of mutations at multiple oncogenes and tumor suppressor genes may be mediated by various signaling pathways.

## MIRS AND HH SIGNALING

miR-326 has been shown to target SMO which inhibits glioma stemness. SMO was determined to be a target of miR-326. Overexpression of miR-326 suppressed SMO levels, Hh pathway activity, decreased self-renewal, stemness, and induced differentiation of U251 tumor cells. Suppression of Hh pathway activity increased miR-326 activity [117].

miR-137 has been determined to be down regulated in glioma stem cells (GSCs) in comparison to neural stem cells (NSCs). The miR-137 promoter region was hypermethylated in glioma specimens. miR-137 targets RTVP-1 (aka glioma pathogenesis-related protein 1) in glioma stem cells and suppresses stemness. The tumor suppressor RTVP-1 was determined to be a critical target of miR-137. Silencing of RTVP-1 decreased GSC self renewal and CXCR4 expression. Overexpression of miR-137 induced neural differentiation in both NSCs and GSCs. Interestingly, expression of pre-miR-137 decreased self-renewal and the expression of Oct4, Nanog, Sox2 and Shh which are stem cell markers [118].

The small molecule Smo inhibitor Erismodegib (NPV-LDE-225) has been shown to have effects on EMT on regulating miR-21, miR-128 and miR-200 expression in glioblastoma cells. Erismodegib inhibited cell viability and induced apoptosis by suppression of miR-21 expression. Erismodegib suppressed the expression of pluripotency factors such as c-Myc, Nanog, Oct4 and Sox2. Erismodegib had effects on other miRs such as induction of miR-128 which suppressed BMI1. Erismodegib inhibited EMT by increasing E-cadherin and decreasing N-cadherin, Slug, Snail and Zeb1 expression. This was postulated to occur via the induction of miR-200 [119].

The Hh pathway is also important in chronic myeloid leukemia (CML) LSCs. Suppression of the Hh pathway depletes CML LSC. Increased Smo activity was associated with reduced miR-326. miR-326 targets Smo in CD34+ CML stem/progenitor cells. Overexpression of miR-326 downregulated both Smo and proliferation in CML CD34+ cells. Thus, miR-326 appears to regulate Smo expression in some cell types and may be a therapeutic target [120].

## NOTCH AND CSCS

Prolonged herceptin treatment of NCI-N87 gastric cancer cells resulted in the recovery of cells that were herceptin-resistant. The resistant cells had some properties of cells having undergone EMT and were more invasive and metastatic. Long term treatment with herceptin inhibited phosphorylation of Akt but activated Stat-3 which resulted in IL-6 expression. In addition, the Notch pathway was activated in the resistant cells, as upregulation of the Notch ligand Jagged-1 and the Notch-responsive genes Hey1 and Hey2 was observed. Suppression of the Notch pathway inhibited IL-6 expression and herceptin-resistance. Likewise, inhibiting Stat-3 signaling eliminated IL-6-induced Jagged-1 expression and herceptin-resistance [121].

**Figure 15:**
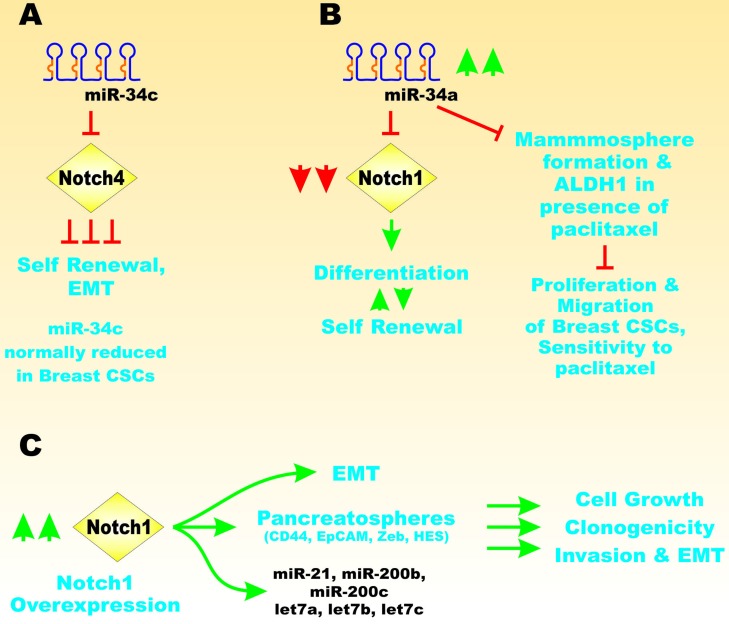
Effects of miRs on Notch Signaling Panel **A**. Ectopic expression of miR-34c can result in suppression of Notch4 expression which blocks self renewal and EMT. Panel **B**. Overexpression of miR-34a can block Notch1 expression and inhibit mammosphere formation, migration, proliferation and drug resistance. Notch1 can serve as a balance between self-renewal and differentiation and is regulated by miR-34a. Panel **C**. Notch1 overexpression can influence EMT and pancreatospheres as well as miRs. These events can influence cell growth, clonogenicity and EMT. This figure is presented to provide the reader an idea of some of the mechanisms that Notch and miRs can regulate important genes involved in self renewal, differentiation and EMT.

miR-34a targets Notch1 in MCF-7 breast cancer cells. Inhibition of Notch1 by miR-34a suppressed proliferation, migration, invasion and breast CSC propagation. Overexpression of miR-34a increased the sensitivity of breast cancer cells to paclitaxel. This was demonstrated to occur by suppression of the Notch1 pathway. Overexpression of miR-34A also reduced mammosphere formation and ALDH1 in the presence of paclitaxel [122]. Figure 15 presents a diagram documenting the effects of miR-34 and Notch on EMT.

miR-134 inhibits proliferation of human endometrial CSCs (HuECSCs). In contrast, the expression of miR-134 was different in human endometrial cancer cells (HuECCS). miR-134 targeted protein O-glucosyltranferase 1 (POGLUT1). Overexpression of miR-134 decreased the expression of POGLUT1 and Notch pathway members in HuECSCs and suppressed xenograft formation [123].

miR-34a has been shown to be a cell-fate determinant in colon CSCs. The levels of miR-34a influence the balance between self-renewal and differentiation of colon CSCs. miR-34a sequestered Notch1 mRNAs [124].

miR34c expression was determined to be reduced in breast tumor initiating cells (BT-ICs) prepared from MCF-7 and SK-3rd breast cancer cells. SK-3rd is a breast cell line which is enriched with BT-ICs. Elevated expression of miR-34c was determined to inhibit the self-renewal and EMT. This occurred by decreasing Notch4 expression [125].

TP53 regulates the expression of miR-34. Expression of miR-34a may be decreased in pancreatic cancer by epigenetic mechanisms as its expression was restored by 5-Aza-dC and SAHA (suberanilohydroxamic acid). These treatments inhibited cell proliferation, self-renewal, EMT and invasion of pancreatic CSCs. In addition, these treatments inhibited expression of BCL-2, cyclin-dependent kinase 6 (CDK6) and SIRT1 that are putative targets of miR-34a. The upregulation of miR-34a by these drugs resulted in acetylated TP53 in the pancreatic CSCs as well as the TP53 target genes: p21^Cip-1^, p27^Kip-1^ and PUMA. When miR-34a was inhibited by the antagomiR, the effects of 5-Aza-dC and SAHA were suppressed. The Notch pathway was inhibited in the pancreatic CSCs by SAHA.SAHA is a histone deacetylase (HDAC) inhibitor. SAHA treatment also resulted in inhibition of EMT and increased E-cadherin and decreased N-cadherin expression. Inducers of EMT such as: Zeb-1, Snail and Slug were inhibited by SAHA treatment. Both 5-Aza-dC and SAHA suppressed pancreatic CSC migration and invasion [126].

Overexpression of Notch-1 in the ASPC-1 pancreatic cancer cell line has been shown to increase cell growth, clonogenicity and invasion and lead to EMT by stimulation of the mesenchymal markers such as ZEB1, CD44, EpCAM and HES1. Notch-1 was also shown to influence miR expression. Notch expression resulted in increased levels of miR-21 and decreased levels of miR-200b, miR-200c, let-7a, let-7b and let-7c. miR-200b re-expression resulted in decreased expression of Zeb1 and vimentin but increased the levels of E-cadherin. Notch-1 overexpression augmented pancreatospere formation and the expression of the CSC markers including CD44 and EpCAM. Genistein treatment was determined to suppress cell growth, clonogenicity, migration, invasion, EMT, formation of pancreatospheres and expression of CD44 and EpCAM. The authors concluded that Notch-1 signaling is important in the EMT phenotype, development of pancreatospheres and CD44 and EpCAM expression. Some of the effects are mediated by miR-200b [127].

The c-Met, Notch-1 and Notch-2 genes have been determined to be direct targets of miR-34a in gliomas. In glioma patient samples, c-Met levels were determined to be inversely correlated with miR-34 levels. c-Met and Notch were observed to be a least partially responsible for the inhibitory effects that miR-34a had on gliomas. Moreover, miR-34a expression could inhibit xenograft formation. miR-34a was also shown to induce glioma stem cell differentiation. [128].

miR-34 has been shown to regulate Notch and Bcl-2 in pancreatic CSCs. The MIA-PaCa2 and BxPC3 pancreatic cancer cell lines contain mutant TP53 genes. When miR-34 expression was restored in these cells, downregulation of Bcl-2 and Notch1/2 occurred as well as decreased clonogenic cell growth, invasion, increased apoptosis, G_1_ and G_2_/M arrest, and sensitivity to chemotherapy and radiotherapy. CD44+/CD133+ MiaPaCa2 cells with down-regulated miR-34 were enriched with tumorsphere-forming and tumor initiating cells. In contrast, in those cells with miR-34 expression restored, the tumorsphere formation and tumor formation were significantly reduced [129].

miR-199b-5p has been shown to target the transcription of HES1 in medulloblastoma cells. HES1 lies downsteam in the Notch pathway. miR-199-5p was shown to regulate negatively the proliferation and anchorage-independent growth of meduloblastoma cells. Overexpression of miR-199b-5p was also shown to block the expression of many genes that are involved in cancer stem cells. miR-199b-5p expression was examined in 61 meduloblastoma patients. miR-199b-5b expression was significantly higher in the non-metastatic than in the metastatic patients. In addition, the patients with high miR-199b expression had a better overall survival. miR-199b expression in metastatic meduloblastoma may be suppressed by epigenetic or genetic alterations [130].

miR-34 has also been shown to be important in gastric cancer tumorspheres. Kato III gastric cancer cells are mutant at *TP53*. Restoration of miR-34 was shown to reduce the expression of Bcl-2, Notch and high-mobility group AT-hook 2 (HMGA2), chemosensitized the cells and inhibited tumorsphere formation [131].

## SUMMARY

GSK-3 is an important enzyme involved in numerous biochemical processes. The activity of GSK-3 is regulated by numerous kinases and phosphatases. GSK-3 can in turn regulate the activity of numerous substrates. GSK-3 activity can be dysregulated by various signal transduction pathways that are themselves aberrantly regulated in cancer often due to genetic and epigenetic mechanisms. Often the substrates of GSK-3 are proteins involved in EMT and CSCs.

Recently, we have summarized the possibility of using various GSK-3 and Wnt-beta catenin inhibitors to treat cancer patients [4]. GSK-3 inhibitors may show potent effects on certain types of solid tumors. However, since GSK-3 can also function as a tumor suppressor, there has always been concern that inhibition of GSK-3 could serve to stimulate the growth of certain tumors. This does not appear to be the case with at least certain types of cancer, as lithium has been used to treat maniac depression/bipolar disorder patients for a long time and increased cancer incidences to not appear to be present in these individuals. A recent search of clinicaltrial.gov does not reveal any current studies with GSK-3 inhibitors and cancer, although GSK-3 inhibitors are being clinically investigated in neurological diseases. Although, there have been some patients filed for the use of GSK-3 inhibitors in certain types of cancer patients.

miRs can also serve to regulate the expression of many genes involved in EMT. Many different genes have been implicated to be important in EMT and cancer. Recently, the histone genes have been shown to be important in EMT in uterine and ovarian carcinosarcomas [132]. The interactions between GSK-3, PI3K/PTEN/Akt/mTORC1, Ras/Raf/MEK/ERK, Wnt/beta-catenin, Hh, Notch and TP53 pathways are clearly having important roles in regulating EMT and CSCs and some of the components may serve as prognostic indicators for various cancer.
